# The Support can Disguise the Catalytic Effect: The Case of Silver on Alumina in Plasma Ammonia Synthesis

**DOI:** 10.1002/cssc.202402778

**Published:** 2025-05-05

**Authors:** Francesco Spadoni, Sofia Perina, Gaia Castellani, Paolo Tosi, Paolo Fornasiero, Vincenzo M. Sglavo, Luca Matteo Martini

**Affiliations:** ^1^ Department of Physics University of Trento Via Sommarive 14 38123 Trento Italy; ^2^ Present address: Department of Circular Chemical Engineering Faculty of Science and Engineering Maastricht University PO Box 616 Maastricht 6200 MD The Netherlands; ^3^ Department of Chemical and Pharmaceutical Sciences Centre for Energy, Environment and Transport Giacomo Ciamician Consortium INSTM Trieste Research Unit and ICCOM‐CNR Trieste Research Unit University of Trieste Via L. Giorgieri 1 34127 Trieste Italy; ^4^ CNR Institute for Plasma Science and Technology 70126 Bari Italy; ^5^ Department of Industrial Engineering University of Trento Via Sommarive 9 38123 Trento Italy; ^6^ INSTM Trento Research Unit Via G. Giusti 9 50121 Firenze Italy; ^7^ CNR Institute of Photonics and Nanotechnologies Via alla Cascata 56/C 38123 Trento Italy

**Keywords:** ammonia syntheses, nonthermal plasmas, plasma catalysis, plasma chemistry, silver

## Abstract

Plasma catalysis combines the high‐energy chemistry of plasma with the speed and selectivity of chemical reactions in catalysis. However, unlike well‐established thermal catalysis, a better understanding of fundamental mechanisms is needed, as evidenced by the contrasting results reported in the literature. One main challenge is that not only the genuine catalytic effect may play a role, but both the support and the catalyst also impact the plasma, complicating the understanding. In this study, exploring the impact of support by comparing a single metal on various substrates made of the same material is focused on. Herein, silver on γ‐alumina is used to investigate ammonia synthesis in N_2_/H_2_ plasma discharges. Beyond confirming the beneficial role of silver in ammonia formation, it is also found that the influence of support is crucial and it is affected by the preparation method. These findings contribute to clarifying the discrepancies in the literature results despite using the same materials.

## Graphical abstract

Support preparation can significantly impact the conversion in plasma catalytic experiments. Herein, two different discharges are studied: a dielectric barrier discharge and a nanosecond repetitively pulsed discharge, with different supports: alumina beads and laboratory‐made alumina monoliths, both loaded with silver. The crucial role of support preparation is investigated and the positive impact of silver is confirmed.

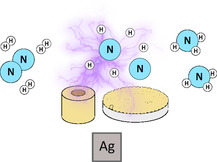

## 1 Introduction

Plasma catalysis is an emerging research area that aims to integrate catalysis processes with a plasma discharge. Undoubtedly, the combination of plasma and catalysis is promising in principle. As a matter of fact, the plasma's high electron temperature produces radicals, ions, and excited states, which can open unconventional reactive pathways. In addition, the presence of a catalyst promotes the conversion of some feedstock into the desired product. The approach offers advantages, such as enabling high‐energy chemistry at low temperatures and creating pathways inaccessible to traditional thermal catalysis. Moreover, plasma sources can directly combine with renewable electricity, assuring low system inertia and allowing the use of intermittent renewable energy sources to convert reactants.^[^ ^1^, ^2^ ^]^ However, many criticalities still prevent understanding the mechanisms underlying plasma catalysis. The reaction pathways within plasma catalysis differ from thermal catalysis and are unique to the specific environment.^[^ ^3^ ^]^ In the thermal process, all the dissociation and recombination reactions occur on the catalyst's surface. Conversely, in plasma, dissociation occurs also in the gas phase. Additionally, the presence of the electric field, charged particles, and radicals can lead to different pathways compared to traditional thermal catalysis.^[^ ^4^, ^5^, ^6^ ^]^

Another aspect to take into consideration is that catalysts need support, which affects the discharge due to both chemical and physical interactions.^[^ ^7^, ^8^, ^9^, ^10^ ^]^ As with the interactions between the plasma and the catalyst, those between the plasma and the support are not well known and are usually considered less relevant. Furthermore, numerous studies have shown that the addition of a catalyst does not necessarily enhance the activity of the plasma, and, in some cases, it can even worsen its performance.^[^ ^7^ ^]^ This behavior has been observed in various reactions, including dry reforming and ammonia production, using a wide range of materials and supports.^[^ ^11^, ^12^, ^13^, ^14^, ^15^ ^]^ These findings point out the need for further investigation in plasma catalysis. It is worth noting that in most plasma catalysis studies, there is no standardized procedure for preparing the catalyst, and sometimes the procedure is only vaguely described. In addition, the methodologies for realizing the reference experiment (i.e., without catalytic material) are often not addressed. This lack of standardization increases the number of variables and can make it challenging to reproduce similar experiments.^[^ ^7^ ^]^ As mentioned earlier, the support is crucial in determining the catalytic effect.

The present study investigates how preparing the support for loading the catalyst affects the catalytic activity. Our approach involves using a support that has undergone the entire preparation process, excluding catalyst loading, as reference support. This choice of reference enables a fair comparison, separating the contribution of the metal from other potential effects due to changes in the support during the preparation process. To our knowledge, this method has yet to be previously explored or specified. In our experiments, we use two different types of discharge (a dielectric barrier discharge, DBD, and a nanosecond repetitively pulsed discharge, NRP) and two different supports (alumina beads and alumina monoliths^[^ ^16^ ^]^). DBD and NRP discharge have been chosen to compare (loaded and unloaded) supports directly interacting with the plasma (DBD) against those that do not directly interact with the discharge (NRP). We choose nitrogen fixation to ammonia as the preferred reaction for several reasons. First, it has already been well studied with nonthermal plasmas.^[^ ^17^, ^18^ ^]^ Additionally, this reaction offers high selectivity for a single product (NH_3_), which simplifies product analysis. This makes ammonia production an ideal case for evaluating the performance of a potential nonthermal plasma catalyst. As metal, we choose silver because computational and experimental findings, for example,^[^ ^19^, ^20^ ^]^ indicate that it could exhibit superior catalytic activity for ammonia synthesis in a plasma environment compared to thermally active metals such as iron and ruthenium.

## 2 Results and Discussion

### 2.1 Characterization of the Metal‐Loaded Support

#### 2.1.1 Beads

To assess the impact of the preparation process on the support properties, we use bare alumina beads as a control group. These beads underwent the same preparation steps as the silver‐impregnated beads (drying, impregnation with or without stirring, and calcination), with the exception that silver is not added. The “impregnation” process (see Catalyst Preparation and Characterization for further details) is conducted using only deionized water. We use this method to establish a reference point and distinguish silver's genuine effect. Additionally, to differentiate the impact of heat treatment from the impregnation step, we create a batch that underwent only the drying and calcination steps, excluding the impregnation process. The batches of alumina beads we prepared are summarized in **Table** **1**.

**Table 1:** Summary of the treatment steps for the different alumina batches.

Batch	Drying	Stirring	Calcination
A	✓	×	×
B	✓	×	✓
C	✓	✓	✓

**Figure 1:**
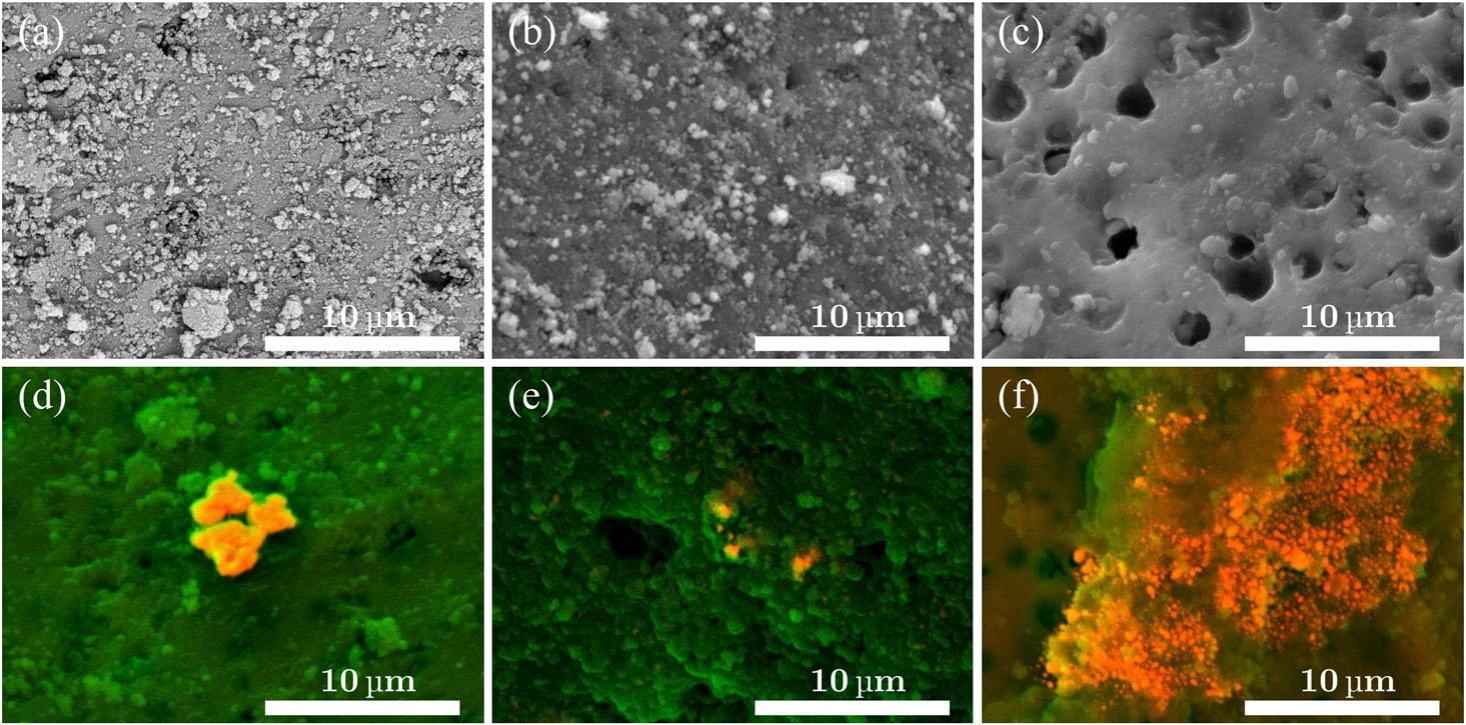
SEM images of a) untreated Al_2_O_3_ beads (batch A) at 5000x, b) thermally only treated Al_2_O_3_ beads (batch B), c) treated Al_2_O_3_ beads (batch C), and d–f) silver‐loaded alumina beads where silver spots and agglomerates of different size and shape can be observed.

Scanning electron microscope (SEM) images of the bare alumina beads, prepared as the control group, were taken for comparison with the untreated beads supplied by the manufacturer. This allows us to observe the effect of our treatment on the morphology of the support. A comparison of the images from batches A and C (**Figure** **1**a,c) demonstrates that our preparation process affects surface roughness. In contrast, when comparing batches A and B (Figure 1a,b), we find that they exhibit similar roughness, suggesting that the thermal processes have little impact on the morphological characteristics of the support.

The silver‐loaded beads were also analyzed with SEM. By combining the images with energy‐dispersive X‐ray spectroscopy (EDXS) analysis, we identified silver spots and aggregates of various sizes and shapes that were lighter in color (see Figure 1d–f). Silver is found even in the darker areas, indicating the presence of smaller, undetectable silver spots. This may be due to limitations in our resolution or the spots being located beneath the surface. Furthermore, the visible spots and aggregates on the surface could result from an excess of silver caused by the saturation of the beads in the concentration range of the solution used during their preparation.

**Table** **2** labels and summarizes the batches loaded with silver, indicating the details of their preparation and the silver surface content obtained with full‐frame EDXS values. In addition, Brunauer–Emmett–Teller method (BET) analysis has been performed on fresh samples (i.e., a fraction of the beads—or a monolith—prepared as for plasma treatment but not exposed to it) and used samples (i.e., a fraction of the beads—or a monolith—used in the plasma for the whole treatment time reported—*vide infra*). BET analysis (see Table S1, Supporting Information) reveals that the mechanical and thermal treatment of the beads does not affect the surface area of the catalyst support, while a surface area reduction between 5% and 10% is ascribable to Ag loading on the support (see fresh batch H and used batches H, I, J, and K). For batches A, B, C, and H, no significative effect of plasma exposure on the surface area is observed. X‐ray diffraction (XRD) patterns of the beads are reported in the Supporting Information (Figure S1 and S2, Supporting Information). All the samples show the predominance of broad reflections related to γ–Al_2_O_3_ in a nanostructured form, in agreement with the very high surface area measured for these samples. No reflections related to α–Al_2_O_3_ can be distinguished in the XRD patterns. No modifications are observed between the fresh and used batches A, B, and C. In the XRD patterns obtained for the fresh batch H, no reflections clearly related to Ag‐based species can be distinguished, indicating that Ag‐containing phases are highly dispersed on the surface of the γ–Al_2_O_3_ support (as suggested by the SEM‐EDXS analysis). After using the loaded beads, very sharp reflections are superimposed on those related to the support. These reflections have been associated to the presence of metallic Ag and AgAlO_2_, suggesting that Ag aggregates on larger spots due to plasma exposure. Increasing the amount of Ag on the beads (batches I, J, and K), it's possible to observe that the intensity of the reflections related to metallic Ag and AgAlO_2_ increases.

**Table 2:** Table summarizing the details of the preparation and the characteristics of the different silver‐loaded batches prepared.

Batch	Solution concentration [wt%]	Ag load [wt%]	Surface Ag [mass norm%]
H	5.04 ± 0.05	1.37 ± 0.01	5.8 ± 0.4
I	2.49 ± 0.03	2.03 ± 0.05	7.6 ± 0.8
J	4.50 ± 0.05	4.40 ± 0.04	7.5 ± 0.6
K	10.2 ± 0.1	9.24 ± 0.05	13 ± 2

#### 2.1.2 Monoliths

Cylindrical γ–Al_2_O_3_ on α–Al_2_O_3_ catalytic supports are specifically designed for the NRP reactor. The primary difference in this configuration compared to that using beads is that the support does not directly contact the plasma but surrounds the area where the discharge occurs.

**Figure 2:**
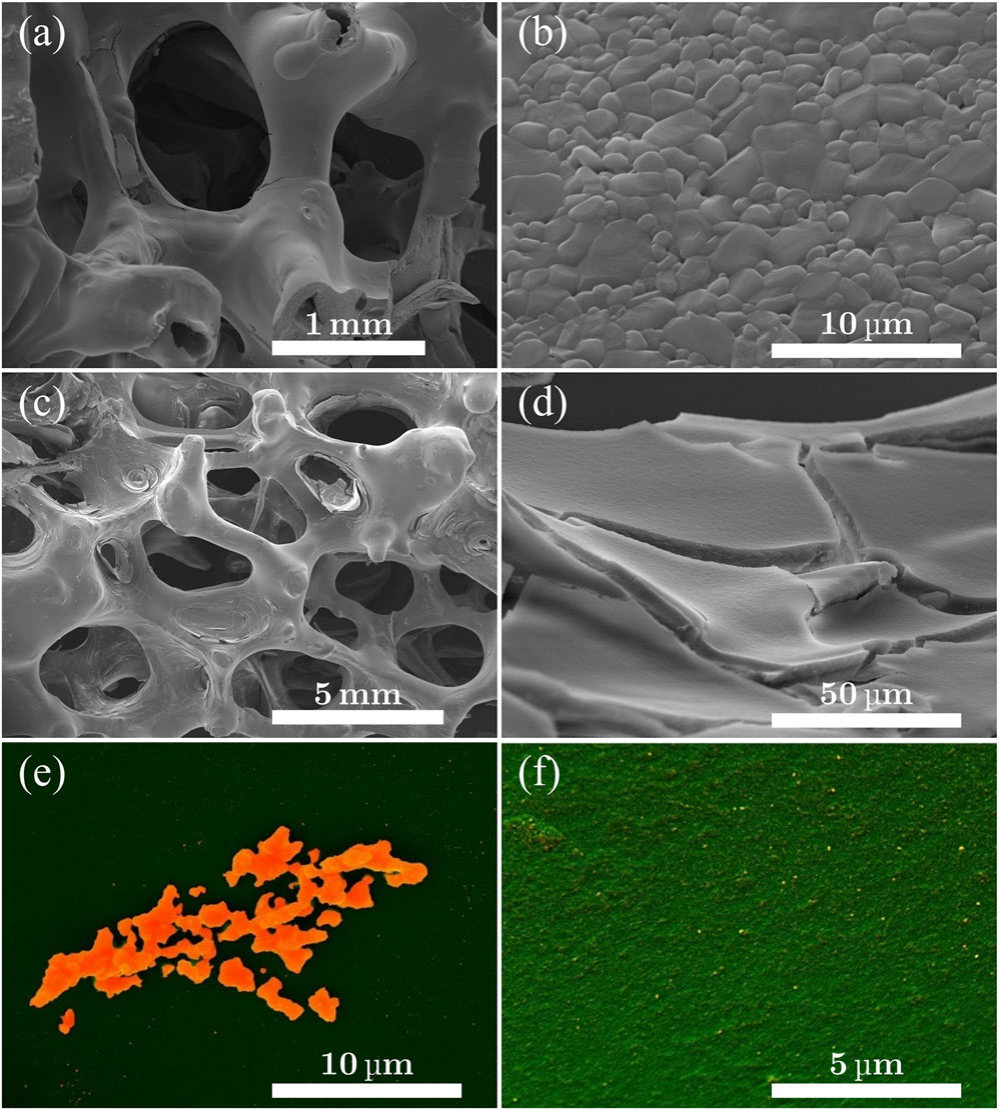
SEM images of the structure of the monoliths. First row: a,b) α‐Al_2_O_3_; second row: c,d) γ‐Al_2_O_3_ layer; and third row: e,f) Ag deposit on γ–Al_2_O_3_.

The macroporous structures and the detailed α–Al_2_O_3_ structure of the bare α–Al_2_O_3_ are presented in **Figure** **2**a,b. The results of the deposition of γ–Al_2_O_3_ phase on top of the α–Al_2_O_3_ structure are shown in Figure 2c,d where it is possible to observe that the deposition is uniform over the α–Al_2_O_3_.

**Figure 3:**
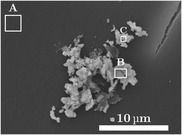
A–C) Areas where the EDXS analysis of silver deposition on γ‐Al_2_O_3_ monolith has been performed. The chemical composition of three different areas is reported in **Table** **3**.

Because of the morphology of the samples, there are some inhomogeneities in the deposition of Ag on γ–Al_2_O_3_ (visible in Figure 2e), but this does not influence the experiment's outcome, as we will show. On average, the Ag uniformly spread on the γ–Al_2_O_3_ as testified by Figure 2f. For 20 PPI (PPI—pores per inch) monoliths, we manage to achieve a +15 wt% in γ‐alumina loading, while for the 30 PPI, we reach +30 wt%. The loading of the silver is set between 2 and 5 wt% with respect to the γ‐alumina. Additional proofs of the efficacy of the deposition of γ–Al_2_O_3_ phase on α–Al_2_O_3_ is inferred from the comparison of XRD patterns of α–Al_2_O_3_ with and without γ–Al_2_O_3_ covering as reported in Figure S4, Supporting Information, where peaks of γ–Al_2_O_3_ are visible together with α–Al_2_O_3_ ones in the γ–Al_2_O_3_‐covered sample. The BET surface area of α–Al_2_O_3_ monoliths is at least two orders of magnitude lower than γ–Al_2_O_3_‐covered monoliths, which show a surface area around 36–67 m^2^ g^−1^ (see Table S2, Supporting Information). We also used EDXS to confirm if a structure is silver or not. Here we report an example in **Figure** **3**. Even if no silver particles are visible, the presence of the metal is still detectable, similar to what is observed with the beads. This indicates that there could be silver particles of the order of tens of nanometers.

**Table 3:** EDXS results of the silver deposition in percentage of atoms in the three different areas highlighted in Figure 3.

	Ag atom [%]
Area A	1.1 ± 0.3
Area B	38 ± 4
Area C	30 ± 3

### 2.2 Ammonia Synthesis in the DBD

#### 2.2.1 Catalyst Support (Alumina)

**Figure 4:**
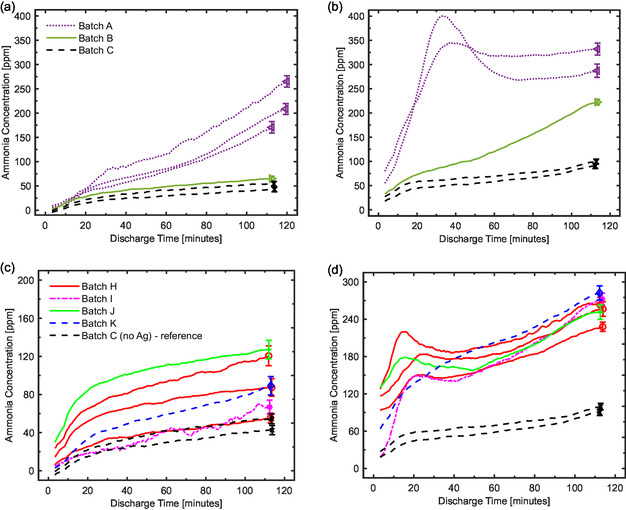
First row: comparison of the NH_3_ production for the a) first use and b) second use of the batches A (untreated alumina), B (calcined alumina), and C (treated alumina), as a function of the discharge time. Second row: comparison of the NH_3_ production for the c) first use and d) second use of the batches H, I, J, and K (silver‐loaded alumina) as a function of the discharge time, where batch C (treated alumina) is the corresponding alumina reference. Lines of the same color represent repetitions of the measurement with different beads from the same batch. The given errors are statistical errors (2*σ*).

The different alumina beads from batches A, B, and C are placed inside the reactor, realizing a packed‐bed configuration. The NH_3_ production for the DBD reactor as a function of the discharge time is reported in **Figure** **4**a,b for the first and second measurements, respectively. To demonstrate the effect of the substrate treatment and Ag addition in the DBD configuration, two consecutive runs are performed, each lasting ≈115 min. While achieving a steady‐state production of ammonia within this timeframe is unlikely, the performance differences among the various configurations are evaluated based on the changing trends of its production over time.

Untreated alumina (batch A) exhibits the highest production in both initial and subsequent uses. This difference is especially significant compared to batch C (treated alumina), although it diminishes in the second use for batch B (calcined alumina). In the first use, batch A does not reach the equilibrium, and NH_3_ production grows up to 250 ppm. Conversely, batches B and C reach a plateau around the 50 ppm level. In the second use, batch A undergoes a desorption process (indicated by the peak before the 40 min discharge time) of previously produced ammonia. Desorption, then, sums with the production process, reaching a steady state around 300 ppm. Batch B, however, continues to grow beyond 200 ppm, getting closer to batch A. Batch C grows slowly up to 100 ppm.

The morphology of the beads in batch A suggests a reason for their higher activity. This effect is likely physical rather than chemical, as the batches differ only in surface properties, not composition. By enhancing the local electric field, we suggest that surface roughness influences discharge characteristics. As for the behavior of batch B during the second use, we propose that the calcination process generates more available free sites on the surface by desorbing most of the molecules from the pores.^[^ ^21^, ^22^ ^]^ Therefore, we observe a behavior similar to the first run of batch A during the second use. This may occur because the beads may not be fully filled with ammonia, as we have not yet observed the desorption curve. This behavior is also suggested by the analysis of temperature‐programmed desorption (TPD) measurements on fresh beads from batches A, C, and H that are reported in Supporting Information (TPD Analysis section). Figure S7, Supporting Information, shows that the mechanically and thermally treated beads desorb more ammonia at higher temperatures compared to the untreated ones. The addition of Ag greatly increases the adsorption of ammonia, suggesting that the ammonia production in the case of Ag‐loaded beads might be even greater in the steady state (i.e., Ag‐loaded beads adsorb more ammonia than unloaded beads).

Our measurements indicate that the performance of the reactor in producing ammonia is significantly influenced by the treatment of the packed catalyst support, which modifies the morphology of the alumina beads. To distinguish the effect caused by the metal from the effect caused by the support, batch C is the appropriate reference to consider. This is because it has the same surface morphology as the silver‐loaded beads.

#### 2.2.2 Silver‐Loaded Support

The NH_3_ production is shown in Figure 4c,d for the first and second use, respectively. In the first use, the silver‐loaded batches performed similarly to, or even better than, the alumina reference. In the second use, this difference was amplified; the increased production of batches H–K compared to batch C can be attributed to an interaction between the plasma and silver. The ammonia concentration does not appear to correlate with the amount of silver loaded on the beads, as evidenced by the results from the second use. Figure 4d shows that after the desorption process—more pronounced for some beads than others—all the different batches (H–K) approach similar values when compared to batch C. This behavior occurs due to the saturation of the beads from absorbing ammonia. We remark that the effect of silver is observed only when batch C is used as a reference. This batch is the alumina support that undergoes the entire treatment except for metal loading.

### 2.3 Ammonia Synthesis in the NRP

**Figure 5:**
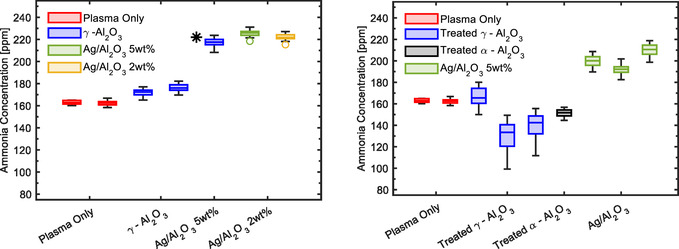
NH_3_ production using 20 PPI (left) and 30 PPI (right) monoliths as supports. The measurements obtained after reaching equilibrium in the NH_3_ production (from 35 to 45 min of NRP discharge) are used to make the box plot. NRP discharge and gas flow parameters: 600 Hz, 150 sccm H_2_ and 50 sccm N_2_. *: non‐calcined monolith.

Two different types of polyurethane foams have been employed for the monolith preparation: 20 and 30 PPI. Results are reported in **Figure** **5** for both 20 (left) and 30 PPI (right). Due to the lower alumina content in the γ phase, we achieved equilibrium during the measurement more quickly compared to the DBD. After 35 min of NRP discharge, the NH_3_ concentration reached equilibrium, and the data collected in the successive 10 min of discharge are presented in the box plots of Figure 5. For both the 20 and 30 PPI foams, we see a 25%–30% increase in the production rate of NH_3_ on average due to the presence of silver. And, 20 PPI goes from 175 to 220 ppm, and 30 PPI goes from 160 to 200 ppm. As for the beads, the production rate does not depend on the amount of silver loaded on the support. In the case of the NRP discharge, the impact of the support's presence on ammonia production appears minimal compared to plasma‐only measurements. All comparison measurements with silver were conducted using γ‐alumina‐coated monoliths that underwent the same procedure as those loaded with silver. In this case, no stirring process was involved; instead, “impregnation” with deionized water and thermal treatment for calcination were conducted.

Figure 5 (left) for the 20 PPI support also shows the measurements using untreated support, clearly showing the difference compared to those that underwent the complete treatment: it is sufficient to conceal the effects of silver. Although the NRP experimental setup differs significantly from the DBD in geometry, discharge intensity, and catalyst placement, the preparation of the support still affects ammonia production. Choosing the appropriate reference is crucial to demonstrate the silver‐enhancing effect.

## 3 Conclusion

In this study, we examined the role of support by comparing a single metal across various substrates made from the same material. The roles of the support and metal have been clarified and better understood.

### 3.1 Role of the Support

Our study confirms that the catalyst support significantly influences the outcome of the experiment, as already noted by several authors. Supports without silver, treated in the same way as those with silver, perform worse than untreated ones. This effect is consistently observed across both supports (beads and monoliths). This difference can be attributed to the combination of stirring (applicable only to beads) and the heat treatment at 550 °C that the supports undergo. Specifically, the heat treatment, which is the only process shared by both beads and monoliths, may activate the alumina sites, enhancing their ability to absorb gas. The magnitude of the effect may differ due to the substantial difference in the amount of γ‐alumina present in the two reactors: ≈3 g in the DBD and hundreds of milligrams in the NRP. The specific roles of certain steps, such as stirring the beads, and the characteristics of the resulting support, such as pore exposure, remain unclear. We have demonstrated that the preparation of the support significantly impacts the final results, particularly when comparing the catalyst's performance to a reference. Choosing the right reference is crucial; if chosen incorrectly, it could obscure the catalytic effect.

### 3.2 Role of Silver

Adding silver to the supports was found to increase the production of NH_3_ in both discharges. This outcome becomes evident only when comparing the performance of silver‐loaded supports to that of unloaded supports processed similarly. The most significant increase in production due to silver is observed in the beads within the DBD during their second use, reaching up to +150%. In the NRP, silver contributes to a notable increase in NH_3_ production when using monoliths, with an increase of +30%. Interestingly, the increase is not significantly affected by the amount of silver loaded onto the supports. This may be due to the supports being already saturated with silver nanoparticles. Any additional silver forms clumps larger than 5 nm, as observed in all samples. It's possible that these larger clumps don't contribute to the enhancement effect.^[^ ^23^, ^24^, ^25^ ^]^ Another crucial observation is that the silver enhancement occurs regardless of the catalyst's position within the plasma region, this effect having been observed in both reactors. To assess whether the increase in ammonia production results from the genuine catalytic effect of silver or from other interactions between the plasma, metal, and supports, further experiments are necessary.

To ensure that findings across studies can be compared and reproduced, it is essential to standardize plasma catalysis experiments. Our research emphasizes the importance of considering the support preparation process when evaluating a metal's activity in a plasma environment. Neglecting this aspect may lead to inconclusive results in plasma catalysis.

## 4 Experimental Section

4.1

4.1.1

##### Experimental Setup

**Figure 6:**
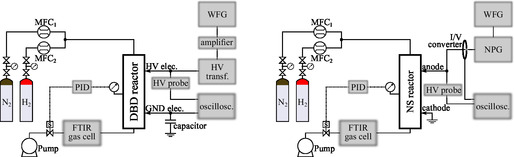
General setup scheme. Left: DBD. Right: nanosecond repetitively pulsed discharge. N_2_ and H_2_: N_2_ and H_2_ gas cylinders, respectively, MFC: mass flow controller, PID: proportional–integral–derivative controller acting on the proportional valve, WFG: waveform generator, HV probe: high‐voltage probe, HV transf.: high‐voltage transformer, NPG: nanosecond pulse generator, HV elec: high‐voltage electrode, GND elec: ground electrode, and oscillosc: oscilloscope.

The general setup for both reactors (DBD and NRP) is reported in **Figure** **6**. Mass flow controllers (1179 A, MKS Instruments) regulated the H_2_ and N_2_ (Linde, 99.999% purity) flows entering the reactors. The output was connected to a fourier‐transform infrared (FTIR) spectrometer (Equinox 55, Bruker) equipped with a 2 m multi‐pass gas cell (Cyclon C2, Specac), allowing for the quantification of NH_3_ in the reactor exhaust. The absolute number density was determined starting from the absorption cross sections from the HITRAN2020 database.^[^ ^26^ ^]^ All the experiments were carried out with a reactor pressure of 1 bar kept constant by a PID controller acting on a proportional valve. For all samples, two measurements in subsequent days were performed.

##### DBD

**Figure 7:**
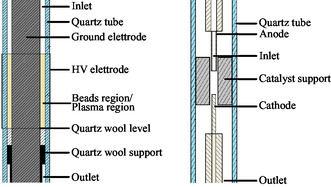
Schemes of the two reactor: left: DBD reactor; right: NRP reactor.

The DBD reactor (**Figure** **7**left) consisted of an internal stainless steel cylindrical electrode (external diameter of 15 mm) connected to ground, a dielectric layer provided by a quartz tube (inner diameter 21 mm, external diameter 25 mm), and an external high‐voltage (HV) electrode made of copper tape coated with tin supported by a Kapton laminate (40 mm length). The catalyst was introduced in a packed‐bed configuration into the reactor by placing its support (alumina beads) in the gap space between the inner electrode and the quartz tube. The alumina that could fit in the discharge region was ≈3 g. To support the beads, we used some quartz wool held by a ceramic support. The HV input was produced using a class D audio amplifier (UCD2k, Hypex Electronics) connected to a HV step‐up transformer (AL‐T1000, Amp‐Line). The signal was generated by a waveform generator (33220A, Agilent). For the electrical characterization of the discharge, an HV probe (P6015A, Tektroniks) and a 1 μF capacitor in series to the ground electrode were used. The voltage input to the DBD reactor was a sequence of bursts, with a frequency of *f*_b_ = 2 Hz and a duration of *T*_p_ = 0.1 s each (resulting in a duty cycle of DC = *T*_p_ *f*_b _= 0.2). The frequency of the sinusoidal wave (*f*_s_) was 10 kHz and its amplitude was (25.0 ± 0.1) kV_pp_.

##### NRP

The NRP reactor (Figure 7right) was made of a quartz cylinder with an inner radius of 20 mm. The electrodes were in a pin‐to‐pin configuration with a 5 mm gap. The anode was a tungsten tube (outer diameter of 3 mm, inner diameter of 2 mm) that provided the gas inlet for the reactor. The cathode was a solid tungsten rod (diameter of 2 mm). The gas exited the reactor through holes located on the cathode support. The catalytic material was placed in a holder in Macor between the two electrodes. The anode was connected by a coaxial cable to the nanosecond power supply (NPG‐18/100 k, Megaimpulse) that delivered pulses with a typical amplitude of 25 kV and 10 ns FWHM. The power supply was operated at a repetition frequency *f*_NRP_ of 600 Hz. An *I*/*V* converter (CT‐D‐1.0, Magnelab) and an HV probe (P6015A, Tektronix) were used to measure the current and voltage signals, respectively.

The input gas flows for both reactors were 50 sccm of N_2_ and 150 sccm of H_2_. The electrical signals were acquired and digitalized with an oscilloscope (HDO9014, LeCroy).

##### Electrical Characterization

The specific energy input was calculated as

(1) $$\text{SEI}\;\;\left[\text{kJ}\;\;{\text{dm}}^{-3}\right]=\frac{P\;\left[\text{kW}\right]}{\Phi \left[{\text{dm}}^{3}\;s^{-1}\right]}$$

(2) $$\text{SEI}\;[\text{eV}\;{\text{molecule}}^{-1}]=\text{SEI}\;[\text{kJ}\;{\text{dm}}^{-3}]\cdot \frac{6.24\times 10^{21}\;[\text{eV}\;{\text{kJ}}^{-1}]\cdot 22.4[{\text{dm}}^{3}\;{\text{mol}}^{-1}]}{6.022\times 10^{23}[\text{molecule}\;{\text{mol}}^{-1}]}$$

where *P* is the dissipated power by the discharge and Φ the gas flow in the reactor. For the DBD, the energy could be calculated starting from the area of the Lissajous figure (A) and considering the frequency of the sinusoidal voltage and the duty cycle of the discharge

(3) $$P_{\text{DBD}}=A\cdot f_{s}\cdot DC$$

**Figure 8:**
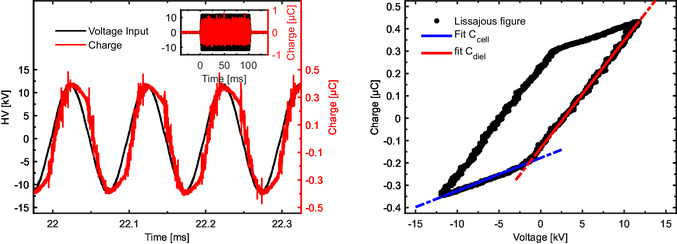
Left: examples of an input burst (blue) and the resulting capacitor response (red) for the DBD reactor. The insert shows an entire burst. Right: Lissajous figure for the DBD discharge. The slopes represent the characteristic capacitances of the reactor (C_diel_: quartz layer capacitance; C_cell_: reactor capacitance).

An example of an input burst and the resulting capacitor response used to determine the charge transferred by the discharge is shown in **Figure** **8** (left), and an example of Lissajous figure in Figure 8 (right). SEI values are presented in **Table** **4**, along with the capacitance values of both the reactor (quartz layer capacitance in series with the discharge gap capacitance) and the dielectric (quartz layer capacitance), derived from the slopes of the Lissajous figures.

**Table 4:** Specific energy input (SEI) results for the three configurations (plasma‐only, catalyst support, and catalyst), as well as the dielectric layer capacitance and reactor's capacitance of the DBD reactor. Errors are statistical (2*σ*).

Sample	SEI [kJ dm^−3^]	SEI [eV molecule^−1^]	Quartz layer capacitance [pF]	Reactor capacitance [pF]
Plasma	2.9 ± 0.5	0.67 ± 0.12	47.0 ± 0.9	8.6 ± 0.6
Al_2_O_3_	3.0 ± 0.4	0.69 ± 0.09	47.8 ± 0.4	12.1 ± 0.7
Ag/Al_2_O_3_	3.0 ± 0.4	0.69 ± 0.09	47.1 ± 0.9	11 ± 1

**Figure 9:**
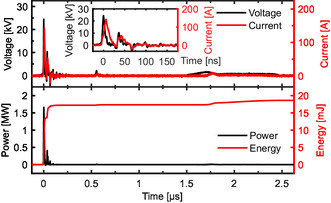
Top: example of voltage input and the resulting current in the NRP reactor with no supports. Bottom: corresponding instantaneous power and dissipated energy.

For the NRP, the voltage and current produced by the HV generator were characterized by an FWHM of about 10 ns and rise time <4 ns on a 75 Ω load. The residual power traveling back and forth in the cable could occasionally reignite the discharge. A typical *I*–*V* trace is shown in **Figure** **9**. From these traces, we could calculate the dissipated instantaneous power and the energy per pulse by integrating

(4) $$P_{\text{NRP}}=f_{\text{NRP}}\cdot \int I_{\text{disp}}(t)\cdot V(t+\delta )dt$$

It was essential to temporarily realign the signals from the HV and current probes. In a no‐discharge regime, the delay (*δ*) was selected so that the time integral of the product *I* × *V* equals zero.^[^ ^27^ ^]^ The results of the SEI determination for the NRP reactor are presented in **Table** **5**. The reactors underwent electrical characterization for the configurations: plasma‐only, with support, and with metal‐loaded support. For both discharges, the specific energy input was set between 3.0 and 3.5 kJ dm^−3^ (0.69–0.81 eV molecule^−1^). A comparison between Lissajous figures in the plasma‐only case (i.e., no catalytic support inserted in the discharge gap of the DBD) and in the presence of the alumina beads is reported in Additional Electrical Analysis section in Supporting Information (see Figure S11, Supporting Information). Additional voltage, current, instantaneous power, and energy traces for the NRP with or without monoliths and with different monoliths are reported in Figure S12, Supporting Information.

**Table 5:** Specific energy input (SEI) for all configurations of the NRP reactor. Errors are statistical (2*σ*).

Sample	SEI [kJ dm^−3^]	SEI [eV molecule^−1^]
Plasma	3.30 ± 0.07	0.77 ± 0.02
20 PPI Al_2_O_3_	3.42 ± 0.08	0.77 ± 0.02
20 PPI Ag/Al_2_O_3_	3.48 ± 0.07	0.79 ± 0.02
30 PPI Al_2_O_3_	3.50 ± 0.07	0.81 ± 0.02
30 PPI Ag/Al_2_O_3_	3.35 ± 0.08	0.78 ± 0.02

##### Ammonia Quantification

The quantity of produced ammonia was derived from the absolute number density in the reactor exhaust from FTIR absorption spectra using absorption cross sections from HITRAN2020 database.^[^ ^26^ ^]^ The transitions chosen for the fit were the ones at 1122, 1141, 1159, 1177, and 1195 cm^−1^. Energy yield for ammonia production could be calculated from the SEI and the ammonia detected in the reactor exhaust. Further details are reported in the Ammonia Production Efficiency section in Supporting Information.

##### Catalyst Preparation and Characterization

The catalytic element used in the experiment was silver. Commercially available γ‐alumina beads (Sasol, Alumina Spheres 1.8/210, nominal diameter 1.8 mm) were used as catalyst support for the DBD reactor, while for the NRP we used in‐house prepared alumina monoliths. The metal‐loaded supports were characterized using SEM, EDXS, XRD, adsorption–desorption experiments analyzed according to the BET and TPD of NH_3_. The Ag load was determined by weighing the supports before impregnation (after vacuum drying to exclude water from the measurement, *m*_dry_) and after calcination (*m*_calc_). The amount of Ag was then calculated as

(5) $$\text{Ag}\;\text{load }[\%]=\frac{m_{\text{calc}}-m_{\text{dry}}}{m_{\text{dry}}}\cdot 100$$

##### Preparation of the Alumina Beads

The alumina beads were placed in a tube furnace (CTF 12/65/550, Carbolite) under vacuum conditions and with an N_2_ flux of 1 dm^3^ min^−1^; the oven was heated up to 130 °C with a ramp of 1 °C min^−1^, then stayed at 130 °C for 4 h. After cooling down to near ambient temperature, the dried beads were removed from the oven and placed in a beaker containing an aqueous solution of silver nitrate salt (AgNO_3_, VWR Chemicals). The concentration of the solution was varied for different batches (from 2% to 10%). The Al_2_O_3_ substrates were impregnated with the AgNO_3_ solution using an autoclave and a membrane pump under vacuum conditions. Then, the solution with the beads was stirred for 1 day, and it was protected from direct light irradiation by covering it with aluminum foil. After the beads completed the impregnation process, they were air‐dried for 2 days. Next, the impregnated alumina spheres were placed in an oven and heated at 1 °C min^−1^ until they reached 130 °C. Once this temperature was reached, they were dried for 4 h. After the drying period, the tube was pressurized to atmospheric pressure without allowing it to cool down. The oven was then heated to 550 °C at 1 °C min^−1^. The beads were subsequently calcined at this temperature for 4 h, using a nitrogen flux.

##### Preparation of the Alumina Monoliths: α–Al_*2*_ *O*_*3*_ Monoliths Preparation

A hollow cylinder of the desired shape and dimension was laser‐cut from polyurethane foam. Foams of different porosity were employed. We used foams with 20 and 30 PPI with dimension: height 20 mm, external radius 18.5 mm, and inner radius 6.5 mm. The cylinder was immersed in a suspension containing 67 wt% α‐alumina powder (Altamis D50), 26 wt% water, 1 wt% dispersing agent (Darvan 821A), and 6 wt% ceramic binder (Duramax B1014). The suspension was mixed using alumina balls in a planetary mixer for 2 h. Afterward, the suspension was filtered through a 40 μm filter, de‐aired, and stirred with an anchor stirrer at 200 revolutions per minute (RPM) for 2 h. The polyurethane foams were soaked in the alumina slurry multiple times, taking care to keep the foam's pores open. To achieve this, we used a gentle blast of compressed air. This process was repeated four times to ensure the alumina slurry thoroughly covers the foams. After soaking, the foams were left to dry at room temperature for at least 2 h before undergoing thermal treatment. The impregnated foams must undergo a furnace treatment up to 1550 °C to completely decompose (by burn‐out) the original polyurethane foam. After the foams were soaked in the alumina slurry, they were left drying at atmospheric pressure at 80 °C overnight. The treatment was composed of the following steps: 1 °C min^−1^, 500 °C for 30 min, 2 °C min^−1^, 800 °C for 30 min, 5 °C min^−1^, 1550 °C for 120 min. Observing a slow increase of temperature up to 800 °C was crucial to ensure the complete and gentle removal of the organics and the foam without creating cracks in the alumina body. The whole process required 16 h and cooling.

##### γ–Al2O3 Coating

The α‐alumina structure was coated in a boehmite dispersion.^[^ ^28^ ^]^ This process resulted in the coating of the structure with an γ‐alumina layer. The dispersion was prepared with 10 wt% of boehmite (DISPERAL P2) in water, along with the addition of 1 wt% of HNO_3_. The mixture was then stirred at high RPM for at least 1 h until it becomes homogeneous. The monoliths are soaked in this solution using the “vacuum impregnation” method, excess solution was allowed to drip off, and the monoliths were then calcined at 550 °C for 1 h. We repeated the impregnation process multiple times until we achieved the desired weight increment. Once the target weight was reached, a longer calcination was conducted for 4 h at 550 °C before the catalyst loading.^[^ ^28^ ^]^

##### Silver Loading

The wet impregnation method using a silver nitrate solution added silver to the support, employing the vacuum impregnation technique described for the beads. The drying process was done in a vacuum using a membrane pump to evaporate the excess water. The monoliths were calcined using the same temperature ramp as that used for the beads. The cylinders were drained of the solution and air‐dried for 2 days. After that, they were placed in an oven and heated at a rate of 1 °C min^−1^ until reaching 130 °C, where they were dried for 4 h. Following this, the pressure in the tube was increased to atmospheric pressure, and the oven was further heated to 550 °C at the same rate of 1 °C min^−1^. The cylinders were then calcined at this temperature for 4 h while maintaining a nitrogen flux of 1 dm^3^ min^−1^.

## Conflict of Interest

The authors declare no conflict of interest.

## Supporting information

Supplementary Material

## Data Availability

The data that support the findings of this study are available from the corresponding author upon reasonable request.
