# Supplementary material for: The Support can Disguise the Catalytic Effect: The Case of Silver on Alumina in Plasma Ammonia Synthesis
Source: ChemSusChem. 2025 May 5;18(13):e202402778. doi: 10.1002/cssc.202402778 (PMC12232085; doi:10.1002/cssc.202402778)
Supplement: Supplementary file 1 — Supplementary Material [file CSSC-18-e202402778-s001.pdf]

Supporting Information of: The Support Can Disguise the Catalytic Effect: the Case of Silver on Alumina in Plasma Ammonia Synthesis

Francesco Spadoni,+,[a] Sofia Perina,+,[a],[1] Gaia Castellani,[c] Paolo Tosi,[a],[b] Paolo Fornasiero,[c] Vincenzo M. Sglavo,[d],[e],[f] Luca Matteo Martini,*[a],[b]

[a] F. Spadoni, S. Perina, Prof. P. Tosi, Prof. L. M. Martini*
Department of Physics, University of Trento, Via Sommarive 14, Trento, 38123, Italy
E-mail: luca.martini.1@unitn.it

[b] Prof. P. Tosi, Prof. L. M. Martini*
CNR Institute for Plasma Science and Technology, Bari, 70126, Italy

[c] G. Castellani, Prof. P. Fornasiero
Department of Chemical and Pharmaceutical Sciences, Centre for Energy, Environment and Transport Giacomo Ciamician, Consortium INSTM, Trieste Research Unit and ICCOM-CNR Trieste Research Unit, University of Trieste, Via L. Giorgieri 1, Trieste, 34127, Italy

[d] Prof. V. M. Sglavo
Department of Industrial Engineering, University of Trento, Via Sommarive 9, Trento, 38123, Italy

[e] Prof. V. M. Sglavo
INSTM, Trento Research Unit, Via G. Giusti 9, Firenze, 50121, Italy

[f] Prof. V. M. Sglavo
CNR Institute of Photonics and Nanotechnologies, Via alla Cascata 56/C, Trento, 38123, Italy

[+] These authors contributed equally.

[1] Current affiliation: Department of Circular Chemical Engineering, Faculty of Science and Engineering, Maastricht University, PO Box 616, Maastricht, 6200 MD, The Netherlands

Contents

1. XRD Analysis 3

2. BET Analysis 4

3. TPD analysis 7

4. Additional SEM and EDXS Analysis 7

5. Ammonia Quantification 9

6. Ammonia Production Efficiency 9

7. Additional Electrical Analysis 10

1. XRD Analysis

XRD patterns of the spherical samples have been acquired using an Empyrean X-ray diffraction platform (Panalytical), with Bragg-Brentano geometry working in theta-theta configuration. The patterns have been acquired using a Ni-filtered K_α radiation. The contribution of $K_{\alpha 2}$ was removed using the HighScore plus software. Spheres were mounted on a 16 mm sample holder, fixed with an adhesive paste and pressed from the top to be aligned at the zero-position position of the diffractometer (as presented in the picture). The sample was spinned (rotation time: $2\,s^{-1}$) to increase the homogeneity of the sample. XRD patterns were acquired in the range 20 – 90° with a step of 0.033° and an acquisition rate of 45 s/°. Ten patterns have been averaged for each sample to increase the signal-to-noise ratio. Phase identification has been performed by the HighScore Plus software using the Crystallographic Open Database (COD, updated May 2024).

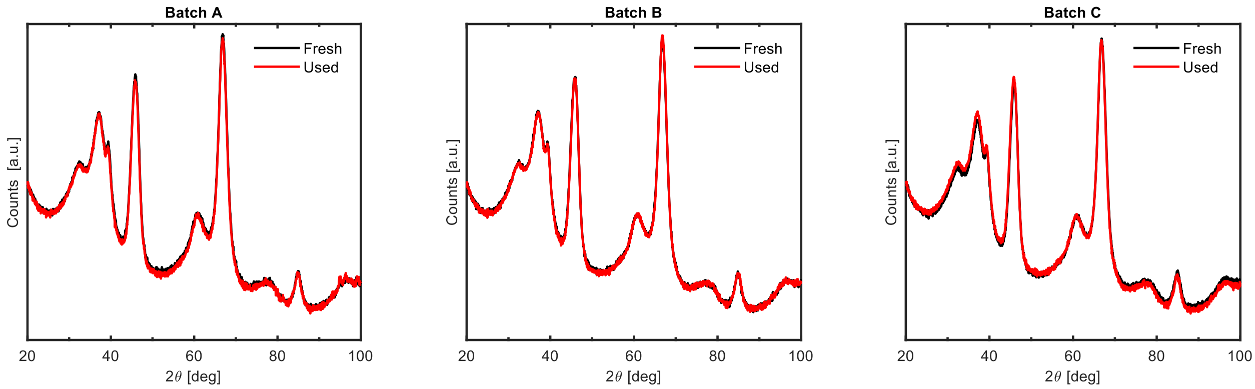

Figure S1. XRD measurements for beads from Batch A, B and C.

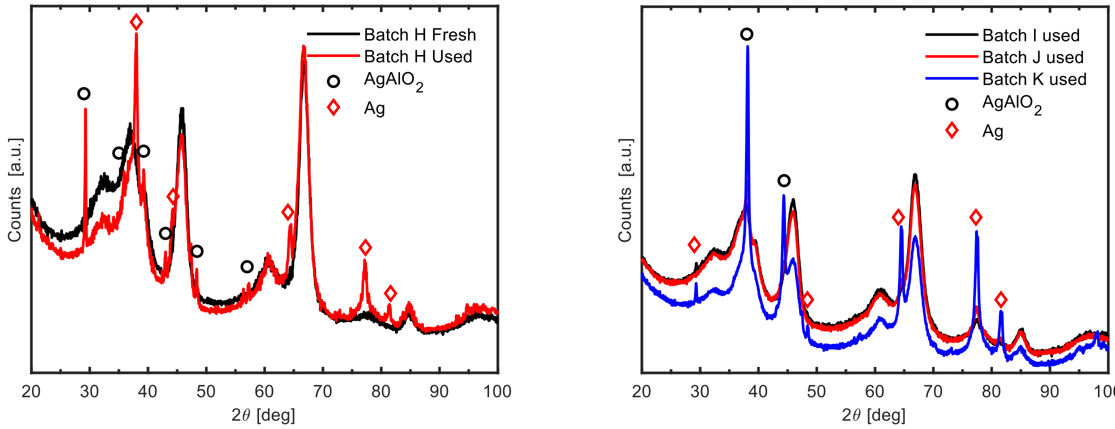

Figure S2. XRD measurements for beads from Batch H, I, J and K.

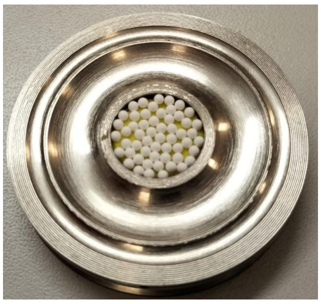

Figure S3. Beads mounted on the XRD sample holder.

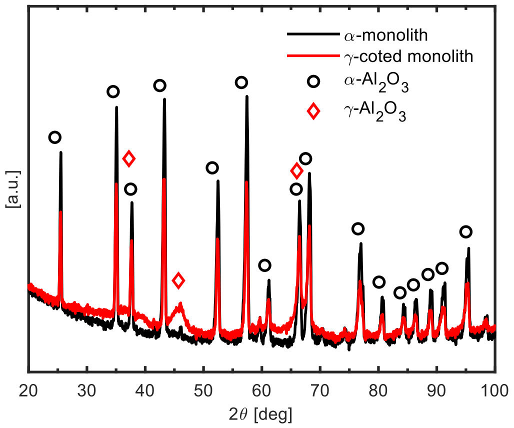

Figure S4. XRD measurements for a γ-Al_2O_3-coated monilith.

2. BET Analysis

Adsorption/desorption experiments were carried out using a 3FLEX Surface Characterization from Micromeritics. Before analysis, the materials were subjected to a two-step degassing procedure. First, a preliminary degassing was performed by heating the samples at 250°C for 2 hours under Ar flow. Subsequently, the samples were in situ degassed at 250°C for 2 hours under a vacuum to ensure the complete removal of adsorbed species. Finally, the adsorption/desorption isotherms were collected at liquid nitrogen temperature (LNT), using N_2 as adsorptive gas for all samples except for samples 15 and 16. Krypton was used as the probe molecule for these latter samples due to their very low surface area. The surface areas of all materials were determined using the BET (Brunauer-Emmett-Teller) model.

Table S1. Beads BET results

Sample	BET surface Area [m^2g^{-1}]
Batch A fresh	203
Batch A used	201
Batch B fresh	200
Batch B used	201
Batch C fresh	204
Batch C used	201
Batch H fresh	188
Batch H used	189
Batch I used	194
Batch J used	192
Batch K used	179

Table S2. Monolith BET results

Sample	BET surface Area [m^2g^{-1}]
20 ppi α-Al_2O_3 fresh	0.4
20 ppi γ-Al_2O_3 fresh	40
20 ppi γ-Al_2O_3 used	36
20 ppi $2wt\%$ $Ag/\gamma - Al2O3$ used	28
30 ppi α-Al_2O_3 fresh	0.04
30 ppi γ-Al_2O_3 used	67
30 ppi $5wt\%$ Ag/γ-Al_2O_3 used	56
30 ppi $5wt\%$ Ag/γ-Al_2O_3 used	52

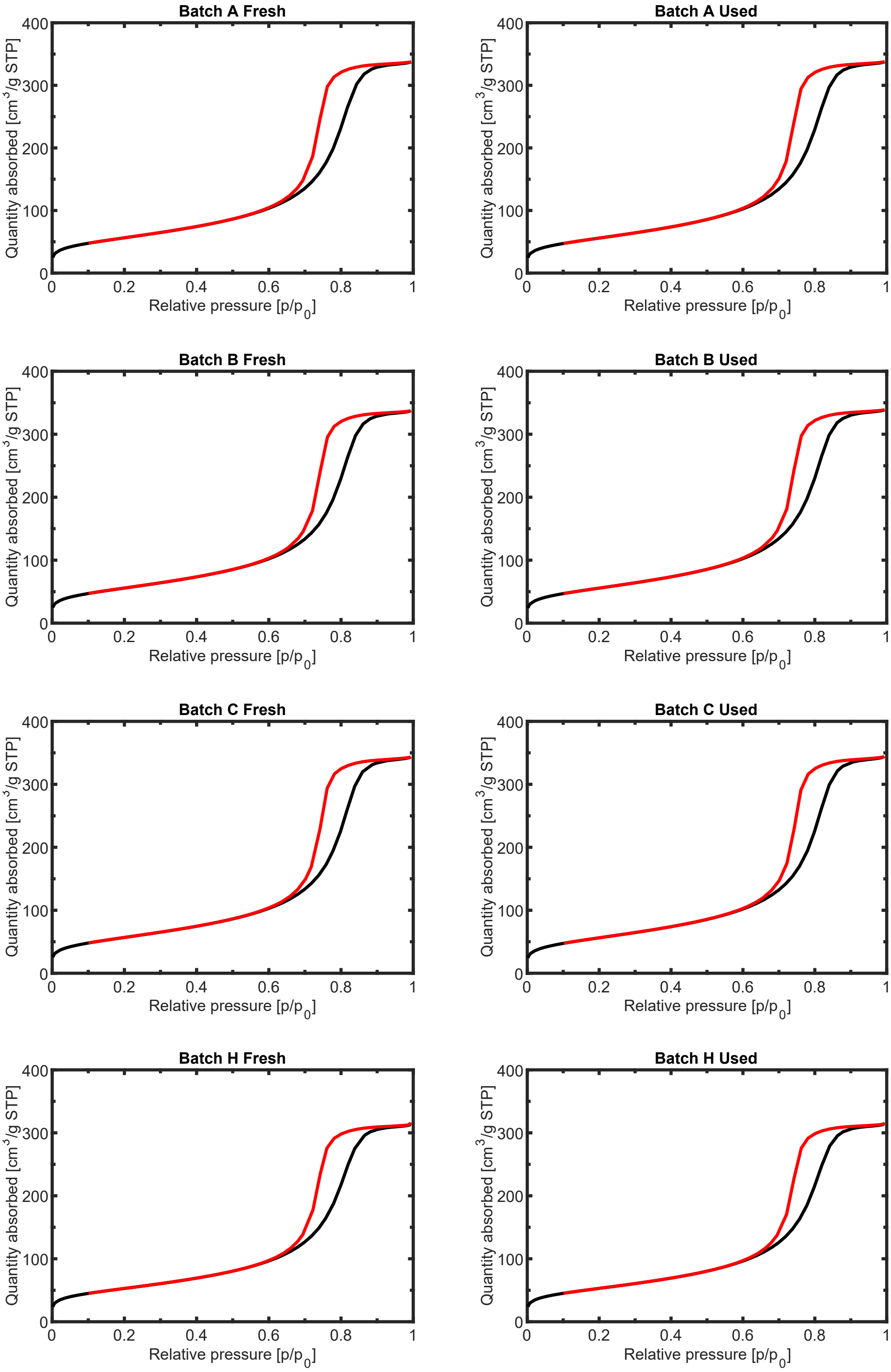

Figure S5. Adsorption (black)/ desorption (red) curves of the BET measurement for beads from batches A, B, C and H.

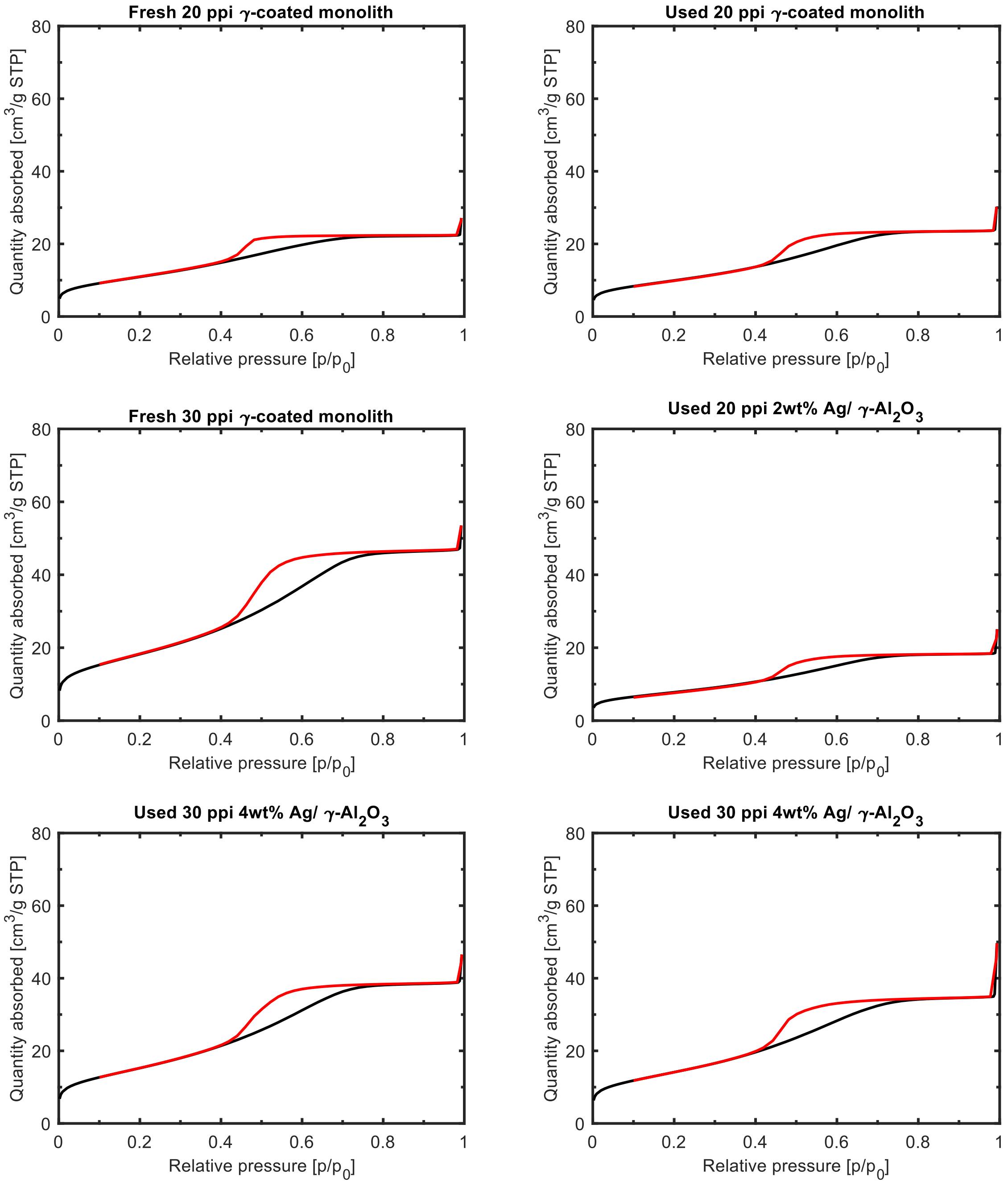

Figure S6. Adsorption (black)/ desorption (red) curves of the BET measurement for different γ-Al_2O_3 and Ag loaded monoliths.

3. TPD analysis

Temperature Programmed Desorption (TPD) where performed by processing 200 mg of beads to temperature programmed oxidation (TPO, 5% O_2 in Ar - 50 mL min^{-1}, 20 °C min^{-1}, 500 °C), NH_3 adsorption (NH_3 - 50 mL min^{-1}, 110 °C, 30 min) and following NH_3 desorption (Ar - 50 mL min^{-1}, 20 °C min^{-1}, 900 °C)

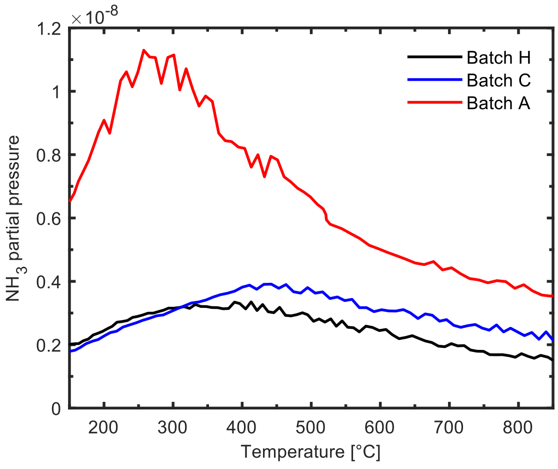

Figure S7. TPD desorption curves.

4. Additional SEM and EDXS Analysis

SEM measurements were performed in a Joel JSM-IT300LV and a Philips XL30 ESEM FEG. EDXS measurements were performed with Bruker Quantax and a XFlash 630M detector.

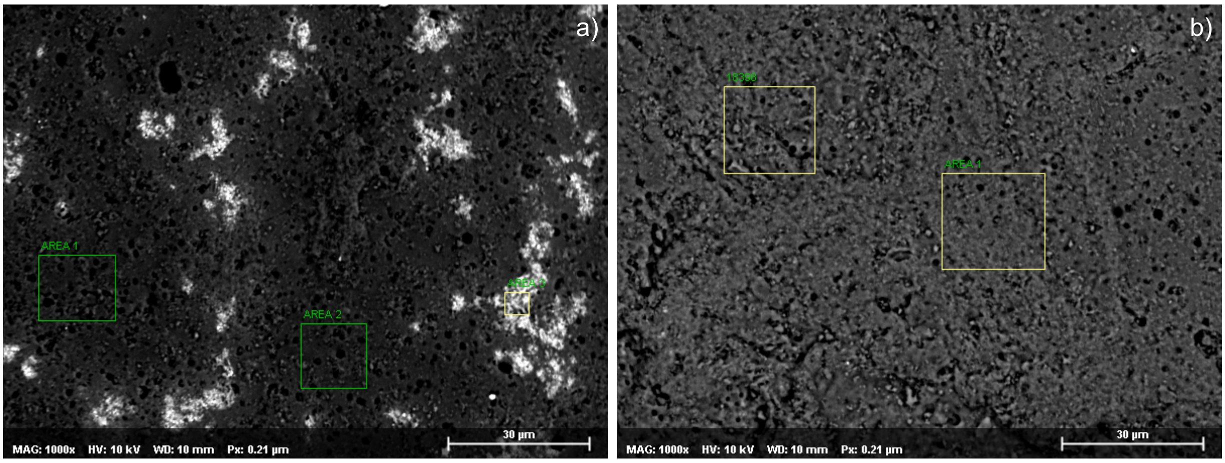

Figure S8. (a) and (b): additional SEM images and EDXS results for Ag-loaded beads.

Table S3. EDXS Results from Figure S8. All errors are 2σ.

(a)	Atom Ag %
Area 1	9.2 ± 0.6
Area 2	8.7 ± 0.5
Area 3	40 ± 3
**(b)**	**Atom Ag %**
Area 1	8.7 ± 0.5
"18398"	8.6 ± 0.5

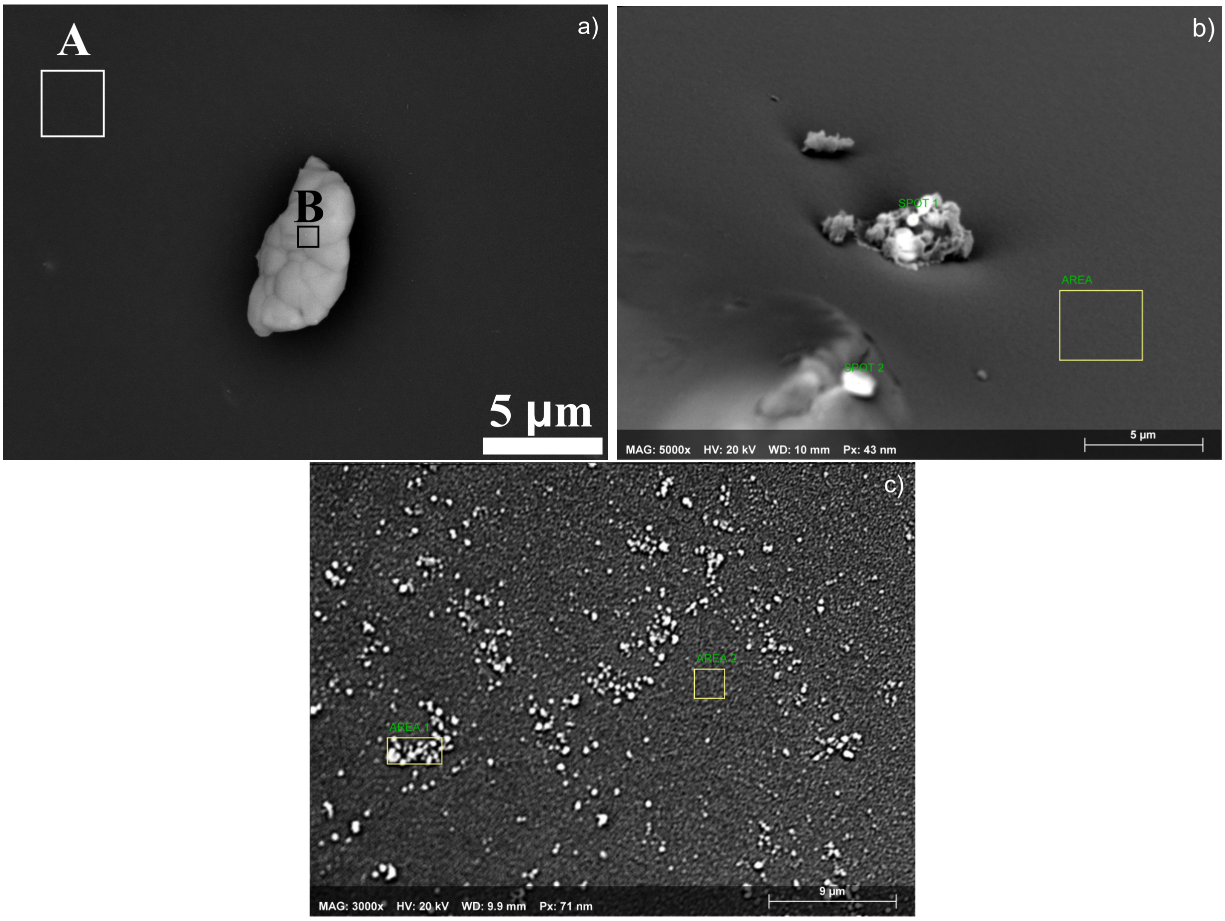

Figure S9. (a) and (b): additional SEM images and EDXS results for different areas of the same monolith presented in Figure 3; (c) SEM images and EDXS results for a different Ag-loaded monolith, the visible spots are particles of gold used for the imaging.

Table S4. EDXS Results from FigureS9. All errors are 2σ.

(a)	Atom Ag %
Area A	1.1 ± 0.2
Area B	44 ± 5
(b)	Atom Ag %
Area	3.3 ± 0.4
Spot 1	30 ± 2
Spot 2	46 ± 3
(c)	Atom Ag %
Area 1	1.1 ± 0.1
Area 2	1.7 ± 0.2

5. Ammonia Quantification

Ammonia absolute number density is determined from the fit of the absorption spectrum in the 1100-1200 cm^{-1} spectral region, corresponding to the ν_2 vibrational mode where the most prominent transitions are at 1103.492 cm^{-1}, 1122.185 cm^{-1}, 1140.708 cm^{-1}, 1159.056 cm^{-1}, 1177.227 cm^{-1}, and 1195.217 cm^{-1} [1]. An example of absorption spectrum is reported in Figure S10-left, while in Figure S10-right a fit over experimental data (with residuals plot) is presented.

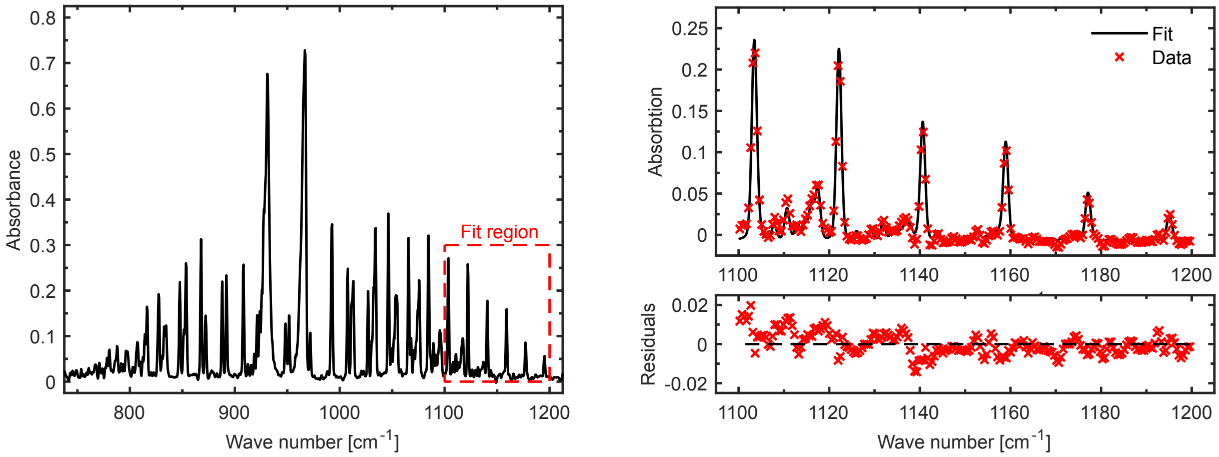

Figure S10. Example of FTIR absorption spectra of NH_3 (left) with a focus on the region between 1100 and 1200 cm^{-1} used to determine the NH_3 absolute number density by the fitting a synthetic spectrum (right).

6. Ammonia Production Efficiency

To compare our results with the literature data, we can compute the energy yield of ammonia production:

$$E_y[g\ kWh^{-1}] = \frac{NH_3\ concentration\ [ppm] \cdot 10^{-6} \cdot\ M_{NH_3}[g\ mol^{-1}]}{SEI\ [kJ\ dm^{-3}] \cdot 22.4[dm^3\ mol^{-1}]} \cdot 3600[s\ h^{-1}] \tag{1}$$

Regarding DBD and NRP discharge, energy yields are reported in Tables S5 and S6, respectively. These values can be compared with literature data [2,3]. Values up to 35.7 g kWh^{-1} in a pulsed DBD with Ru-Mg/γ-Al_2O_3 as catalyst (N_2:H_2 ratio equal to 4:1) have been reported [4]. Literature data on nanosecond discharges are limited, and recent studies on µs-duration discharges report energy efficiency of around 0.20 - 0.28 g kWh^{-1}.

Table S5. Energy yield for DBD configuration. Errors reflect the statistical error (expressed as a two-sigma error).

Sample	Energy Yield [g kWh^{-1}]
Batch A	0.27 ± 0.03
Batch B	0.21 ± 0.02
Batch C	0.07 ± 0.02
Batch H-K	0-19 - 0.26 ± 0.03

Table S6. Energy yield for NRP configuration. Errors reflect the statistical error (expressed as a two-sigma error).

Sample	Energy Yield [g kWh^{-1}]
Plasma	0.13 ± 0.01
20 ppi γ-Al_2O_3	0.13 ± 0.02
30 ppi γ-Al_2O_3	0.12 ± 0.03
20 ppi Ag/γ-Al_2O_3	0.17 ± 0.02
30 ppi Ag/γ-Al_2O_3	0.16 ± 0.02

7. Additional Electrical Analysis

Additional Lissajous figures comparing the electrical behaviour of the DBD with and without beads in the discharge area are presented (Figure S11). A comparison between voltage, current, istantaneous power and energy as a function of time for the NRP configuration in the absence of monolith (Figure S12-g), with α-Al_2O_3 monoliths (Figure S12-a and b), with γ-Al_2O_3 monoliths (Figure S12-c and d), and with Ag/γ-Al_2O_3 monoliths (Figure S12-e and f).

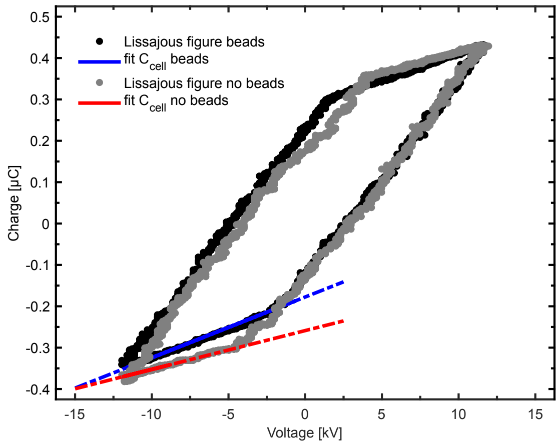

Figure S11. Lissajous figures comparing the electrical behaviour of the DBD with and without beads in the discharge area. The blue and red lines represent a fit of the bottom segment of the Lissajous figures to highlight the difference in the current-voltage derivative (i.e., of the capacitance of the dielectric of the DBD).

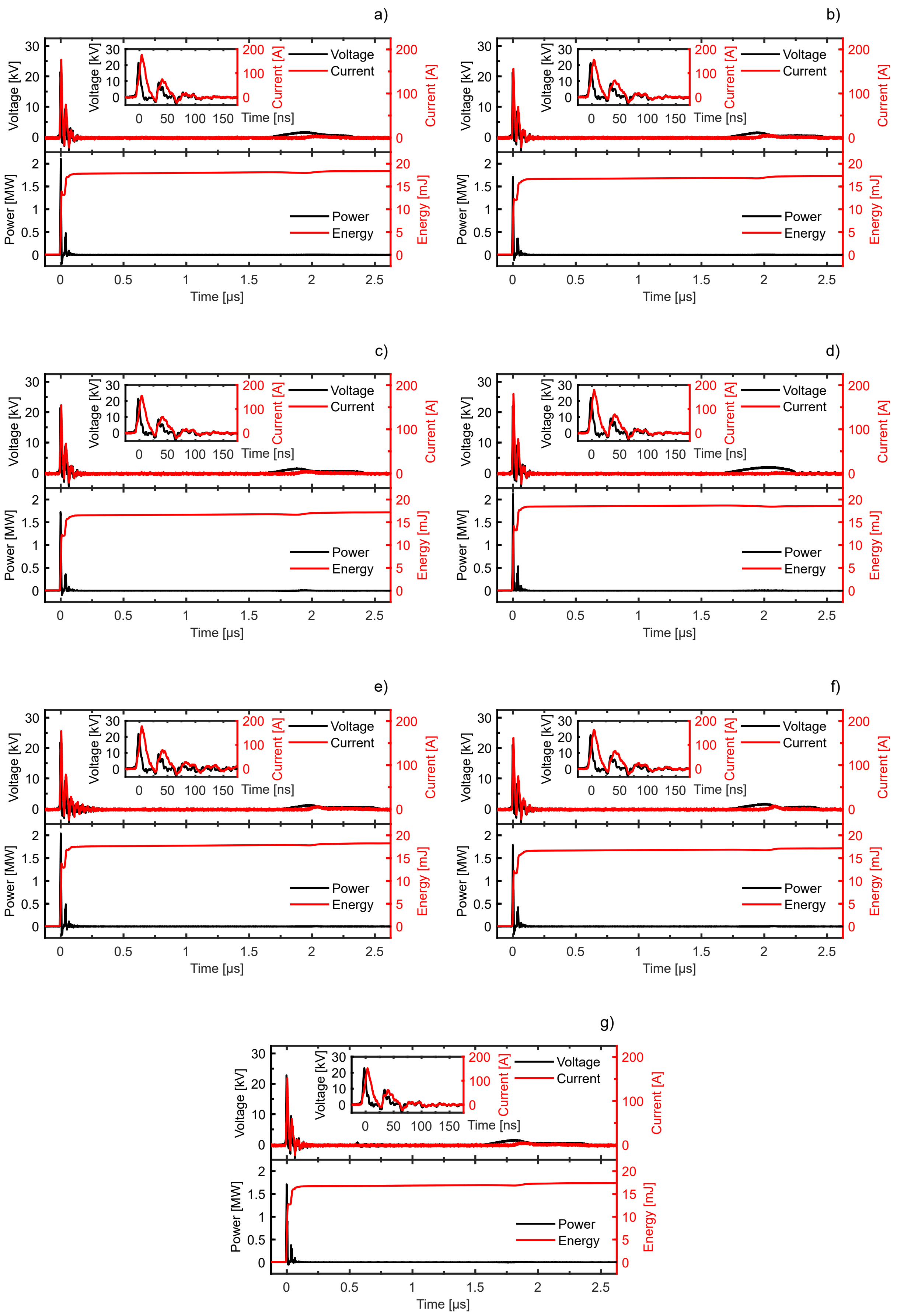

Figure S12. a) 20 ppi α-Al_2O_3 monolith b) 30 ppi α-Al_2O_3 Monilith c) 20 ppi γ-Al_2O_3 monolith d) 30 ppi γ-Al_2O_3 monolith e) 20 ppi Ag/γ-Al_2O_3 monolith f) 30 ppi Ag/γ-Al_2O_3 monolith g) empty reactor - only plasma.

References

[1] C. W. David, *Journal of chemical education* **1996**, *73*, 46.
[2] V. S. Gharahshiran, Y. Zheng, *Journal of Energy Chemistry* **2024**.
[3] J. Zhang, X. Li, J. Zheng, M. Du, X. Wu, J. Song, C. Cheng, T. Li, W. Yang, *Energy Conversion and Management* **2023**, *293*, 117482.
[4] H.-H. Kim, Y. Teramoto, A. Ogata, H. Takagi, T. Nanba, *Plasma Processes and Polymers* **2017**, *14*, 1600157.
